# Sex-specific effects of *guanosine monophosphate synthase* and *steroid receptor coactivator* gene polymorphisms on cashmere fineness and production traits in Liaoning Cashmere goats

**DOI:** 10.14202/vetworld.2026.992-1009

**Published:** 2026-03-15

**Authors:** Qingyu Yuan, Qiying Zhan, Ran Duan, Yichao Zhao, Hao Lin, Shuaitong Li, Weihang Hong, Hua Ma, Lingchao Kong, Wangshu Li, Haoran Wang, Xiaochen Kou, Dakun LYU, Yunlong Guo, Jiamei Liang, Zeying Wang

**Affiliations:** 1 College of Animal Science and Veterinary Medicine, Shenyang Agricultural University, Shenyang 110866, China; 2 Department of Physics, School of Physical Science and Technology, Inner Mongolia University, Hohhot 010021, Inner Mongolia Autonomous Region, China

**Keywords:** cashmere fineness, Cashmere goat, Cashmere production traits, cashmere yield, GMPS gene, Liaoning Cashmere goat, marker-assisted selection, SRC gene

## Abstract

**Background and Aim:**

The *Liaoning Cashmere goat* (LCG) is a dual-purpose breed of major economic importance in China, valued for its high cashmere yield and meat quality. Cashmere fineness (CF) remains a primary target for genetic improvement because fiber diameter directly determines textile value and market price. This study examined the sex-specific effects of single-nucleotide polymorphisms in the *guanosine monophosphate synthase* (*GMPS*) and *steroid receptor coactivator* (*SRC*) genes on CF and a broad spectrum of production traits to identify functional markers for marker-assisted selection.

**Materials and Methods:**

A total of 1,160 healthy LCGs (89 bucks and 1,071 does, 2–4 years of age) from the same nucleus herd were included. The C31799T locus in *GMPS* and the C34197G locus in *SRC* were genotyped by polymerase chain reaction (PCR) amplification followed by bidirectional Sanger sequencing. Genotype–trait associations were tested using general linear mixed models, with genotype, sex, and age as fixed effects, and pedigree information incorporated to control for relatedness. Haplotype phases were inferred separately for each sex with the SHEsis platform. Phenotypes recorded comprised cashmere production traits (fineness, length, yield), body size measurements, slaughter performance, meat quality attributes, milk composition, and lambing rate. Data normality, homoscedasticity, and multicollinearity were verified prior to analysis; statistical significance was declared at p < 0.05.

**Results:**

The TT genotype at the *GMPS* C31799T locus and the CG genotype at the *SRC* C34197G locus were significantly associated with finer cashmere fibers (p < 0.05). Sex-stratified analyses showed that the *GMPS* TT genotype conferred superior CF in does (p < 0.01) and longer staple length in both sexes (p < 0.01), whereas the *SRC* CG genotype improved fineness specifically in does (p < 0.01). Haplotype analysis identified CCCC as the optimal combination for finer cashmere in bucks and TTGG in does. Pleiotropic effects were evident: the *GMPS* CC genotype favored larger body dimensions, the *GMPS* TT genotype enhanced carcass traits in bucks, and the *SRC* CG genotype improved lactation performance. CF exhibited positive correlations with cashmere yield (bucks: r = 0.412; does: r = 0.384; p < 0.01) and negative associations with several slaughter traits. Path and stepwise regression analyses clarified direct and indirect effects, underscoring sex-dependent genetic trade-offs between fiber quality and meat production.

**Conclusion:**

This is the first study to establish *GMPS* and *SRC* as key candidate genes influencing CF in goats. The identified superior genotypes and haplotypes provide sex-specific molecular markers that can be immediately deployed in marker-assisted selection programs to accelerate genetic gains in cashmere quality while safeguarding meat production potential in LCG breeding. Validation in independent populations will further strengthen their utility for precision breeding.

## INTRODUCTION

Cashmere, hailed as the “gem of fibers” [1], is a precious textile material, and cashmere goat farming constitutes a vital economic foundation for many regions. The *Liaoning cashmere goat* (LCG) is a premium breed independently developed in China and is renowned for its stable genetic performance, high cashmere yield, and superior fiber quality [2]. With increasing market demand for high-quality cashmere, fiber diameter (fineness) has become a central metric for evaluating both quality and commercial value. However, the average fiber diameter of LCG remains moderate, not consistently meeting the premium standard of less than 16 μm. Consequently, the continuous reduction of fiber diameter through targeted genetic breeding strategies represents a critical direction for enhancing the breed’s industrial competitiveness. Although cashmere growth is regulated by a complex interplay of genetic, nutritional, and environmental factors [3], the key genetic determinants and underlying molecular mechanisms of cashmere fineness (CF) remain incompletely understood. This knowledge gap substantially impedes the implementation of efficient genetic improvement via marker-assisted selection (MAS).

*Steroid receptor coactivator* (*SRC*) significantly enhances the transcriptional activity of various steroid hormone receptors and plays a key regulatory role in numerous physiological processes, including reproduction and metabolism. It is essential to distinguish between *SRC* coactivators and *SRC* family kinases (SFKs), which belong to two functionally distinct protein families. The former functions as a nuclear transcriptional coactivator, whereas the latter are cytosolic non-receptor tyrosine kinases primarily involved in intracellular signal transduction, including regulation of vascular tone and macrophage-mediated immune responses [4–6]. Both protein classes may participate in the regulation of hair follicle function through distinct mechanisms: the *SRC* coactivator may modulate the proliferation and differentiation of hair follicle stem cells by amplifying sex hormone signals (e.g., estrogen, progesterone), whereas SFKs might indirectly influence hair follicle development and fiber synthesis by affecting perifollicular microvasculature contraction (thereby modulating blood and nutrient supply) or by regulating the local immune microenvironment. The present study focused specifically on the *SRC* gene encoding a nuclear coactivator.

Sex hormones represent the core regulators of hair follicle development and cycling. The *SRC* coactivator interacts with steroid hormone receptors, such as the progesterone receptor (PR), and is a critical component of hormone-dependent transcriptional regulation. Based on this mechanism, we hypothesize that *SRC* may similarly amplify local hormonal signals within skin follicles to coordinate the gene expression network associated with follicle development. This process could ultimately affect secondary follicle density as well as the proliferation and differentiation states of keratinocytes, thereby regulating cashmere fiber diameter. Moreover, *SRC*-mediated hormonal signaling pathways typically exhibit pronounced sexual dimorphism, providing a strong biological rationale for performing sex-stratified analyses of genotype effects.

*Guanosine monophosphate synthase* (*GMPS*) is a key rate-limiting enzyme in the de novo purine synthesis pathway. It catalyzes the amination of xanthosine monophosphate (XMP) to form guanosine monophosphate (GMP), thereby supplying essential nucleotide substrates required for cellular proliferation [7, 8]. During the anagen growth phase, follicular keratinocytes undergo rapid proliferation and intense keratin synthesis, processes that are heavily dependent on an adequate supply of purine nucleotides. Variations in GMPS activity or expression levels may influence cashmere fiber diameter and growth rate by modulating follicular cell proliferation, positioning *GMPS* as a potential metabolic hub for these traits. Early functional studies of *GMPS* in mammalian systems primarily investigated its role in a complex containing *TRIM21 and USP7*, through which it regulates *p53* protein stability and participates in the DNA damage response and the maintenance of genomic integrity [9–12]. The *p53* pathway is also involved in regulating cell cycle progression and apoptosis, suggesting that *GMPS* indirectly contributes to uniform cashmere fiber growth by influencing follicular cell cycle dynamics. Recent advances in tumor metabolism research have demonstrated the significant involvement of *GMPS* in the progression and modulation of the immune microenvironment in various malignancies, including melanoma, liver cancer, and breast cancer [13–15], highlighting its multifaceted biological functions beyond its canonical enzymatic role. The *GMPS* protein contains two functional domains, a glutamine amidotransferase (GATase) domain and an adenosine triphosphate (ATP) pyrophosphatase (ATPPase) domain, that function cooperatively to complete the catalytic reaction [16]. This structural organization implies that genetic variation may precisely modulate its enzymatic function.

Although high-throughput sequencing technologies have accelerated the identification of genetic markers linked to fiber traits across diverse cashmere goat populations, the roles of polymorphisms in the *GMPS* and *SRC* genes in regulating CF and associated production traits remain largely unexplored, especially in the LCG. Prior proteomic screening in LCG highlighted *GMPS* and *SRC* as promising candidate genes for fiber diameter control, yet no systematic studies have characterized their specific single-nucleotide variants, quantified their associations with CF, staple length, yield, body conformation, carcass performance, meat quality or reproductive traits, or investigated potential pleiotropic and sex-dependent effects in this breed. The *SRC* gene, encoding a nuclear transcriptional coactivator that amplifies steroid hormone signaling, is particularly relevant because sex hormone pathways exhibit clear sexual dimorphism in hair follicle cycling and fiber morphogenesis, yet sex-stratified analyses of *SRC* variants have not been conducted in cashmere goats. Similarly, *GMPS*, as the rate-limiting enzyme in de novo purine biosynthesis, is critical for supplying nucleotide precursors during the rapid keratinocyte proliferation of the anagen phase, but its genetic variation and contribution to fiber diameter uniformity in ruminant fiber follicles have received minimal attention. This absence of data creates a significant knowledge gap regarding the molecular mechanisms underpinning CF in LCG, hinders the development of precise, sex-specific MAS protocols, and limits the ability to simultaneously improve fiber quality while maintaining meat production efficiency in this dual-purpose breed.

The present study was therefore designed to address this gap through a comprehensive evaluation of the sex-specific effects of selected single-nucleotide polymorphisms (SNPs) in *GMPS and SRC* on an extensive panel of production traits in a large LCG nucleus herd. Specifically, the objectives were (i) to genotype the C31799T locus in *GMPS* and the C34197G locus in *SRC* by polymerase chain reaction (PCR) amplification and bidirectional Sanger sequencing in 1 160 healthy animals (89 bucks and 1 071 does, 2–4 years of age); (ii) to quantify genotype–trait associations for CF, staple length, yield, body size measurements, slaughter performance, meat quality attributes, milk composition and lambing rate using general linear mixed models that incorporated genotype, sex and age as fixed effects and pedigree information to control for relatedness; (iii) to infer haplotype phases separately for each sex with the SHEsis platform and identify optimal allelic combinations; (iv) to dissect direct and indirect genetic effects through path analysis and stepwise regression modeling; and (v) to propose superior genotypes and haplotypes as immediately deployable molecular markers for MAS. In addition, the study tested three working hypotheses: (1) *GMPS* variants influence fiber diameter by modulating purine nucleotide supply to follicular keratinocytes during the anagen growth phase; (2) *SRC* variants exert pleiotropic effects on growth and metabolic traits in a sex-dependent manner owing to its role as a steroid hormone coactivator; and (3) combined haplotypes of the two loci may produce synergistic regulation of fineness and slaughter characteristics. By establishing *GMPS* and *SRC* as key candidate genes for CF in LCG, the work aimed to provide a robust foundation for precision breeding programs that simultaneously enhance fiber quality and safeguard meat production potential.

## MATERIALS AND METHODS

### Ethical approval

All procedures involving live animals in this study were reviewed and approved by the Laboratory Animal Management and Use Ethics Committee of Shenyang Agricultural University (Approval No. 20240513). The experiment was conducted in full compliance with the institutional guidelines for the care and use of experimental animals and the relevant national regulations on animal welfare in China (including the Regulations on the Administration of Laboratory Animals and the Guidelines for the Ethical Review of Laboratory Animal Welfare).

The study was performed at the LCG Breeding Center, Liaoyang City, Liaoning Province, China. All blood sample collections were carried out under the direct supervision of a licensed veterinarian to ensure minimal stress and discomfort to the animals. No invasive surgical procedures were performed, and all handling was conducted according to the principles of the 3Rs (Replacement, Reduction, and Refinement). The animals remained under standard farm management conditions throughout the study, and no additional pain, suffering, or distress beyond routine husbandry practices was imposed.

Written informed consent for the use of the animals was obtained from the farm management prior to the commencement of the study. The authors confirm that the research adheres to the ARRIVE 2.0 guidelines (Animal Research: Reporting of *In Vivo* Experiments) for the reporting of animal studies.

### Study period and location

This study was conducted from April to August 2025 at the LCG Breeding Center, Liaoyang City, Liaoning Province, China (approximate coordinates: 41.24° N, 123.14° E).

### Animals, inclusion criteria, and management conditions

A total of 1,160 clinically healthy LCG were enrolled in the study (89 bucks and 1,071 does; age range 2–4 years). All animals belonged to the same nucleus breeding herd and were therefore at a comparable production stage. Inclusion criteria were as follows: clinically healthy appearance, absence of major parasitic infestations, and no history of antibiotic treatment within the preceding 4 weeks. Animals were maintained under semi-intensive management conditions with a stocking density of approximately 15–20 goats per pen. They had *ad libitum* access to a total mixed ration and clean drinking water. To maximize genetic diversity within the sampled population, individuals were deliberately selected from multiple unrelated families.

### Phenotypic trait recording and definitions

All phenotypic measurements were performed by the same fixed team of five trained technicians to ensure maximum consistency and to minimize inter-observer variation. Detailed definitions, measurement timing, units, and protocols for each recorded trait are presented in Supplementary Table S1. The main instruments and corresponding calibration procedures are described below.

### Measurement of cashmere production traits and instrument calibration

CF (fiber diameter) and staple length were determined using the portable all-weather CF and length analyzer (Model: OFDA2000, BSC Electronics, Ardross, Australia). The instrument was calibrated weekly before each measurement session using a certified standard cashmere bundle (nominal fineness 15.0 ± 0.1 μm) according to the Chinese national standard Test Method for Cashmere Fiber (GB/T 18267-2019). The calibration deviation was maintained at ≤ 0.2 μm throughout the study. Collected cashmere samples were evenly spread on a dedicated sample slide, the slide was inserted into the detection chamber, and the “Start Test” function was activated to record fiber diameter and length.

### Measurement of body size traits and instrument calibration

Body conformation measurements were obtained using an intelligent three-dimensional optical body measurement system (3D Optical Body Scanner, Model: VITUS Smart, Human Solutions GmbH, Kaiserslautern, Germany). Animals were gently guided into the measurement zone where the system automatically initiated multi-angle scanning. Recorded parameters included: body height, withers height, body length, chest depth, chest width, hip width, heart girth, cannon circumference, and pin bone height. The instrument was calibrated monthly using a standard reference mannequin, achieving a measurement error of ≤ 0.5 cm.

### Measurement of milk composition and instrument calibration

Milk composition was analyzed using the MilkoScan FT120 milk analyzer (Foss Electric A/S, Hillerød, Denmark). The following parameters were determined: milk fat, milk protein, lactose, urea nitrogen, non-fat solids, total solids, conductivity, and health index. Daily calibration was performed before each analysis session using Foss calibration milk (Product No.: 8011-0001, Foss Electric) according to ISO 9622:2013. Calibration error for the major components (fat, protein, lactose) was maintained at ≤ 0.05%. The built-in conductivity sensor was simultaneously calibrated (measurement range 0–20 mS/cm; accuracy ±0.05 mS/cm).

### Blood collection and storage

Whole blood was collected from the jugular vein into commercial ethylenediaminetetraacetic acid vacuum blood collection tubes (Sangon Biotech, Shanghai, China). Immediately after collection, tubes were placed on ice, transported to the laboratory within 4 hours, and stored at –20 °C until DNA extraction (maximum storage duration 2 weeks).

### Extraction and quality assessment of genomic DNA

Genomic DNA was extracted from 200 µL of anticoagulated whole blood using the Ezup column animal genomic DNA extraction kit (Cat. No. B518253-0100, Sangon Biotech, Shanghai, China) following the manufacturer’s protocol. In brief: Proteinase K and lysis buffer (Buffer DL) were added, followed by incubation at 56°C for 10 min. After addition of absolute ethanol and thorough mixing, the lysate was transferred to the adsorption column. The column was washed sequentially with GW solution and Wash solution (two washes each), followed by a high-speed centrifugation step to remove residual Wash buffer. DNA was finally eluted with Capillary Electrophoresis Buffer. DNA purity and concentration were assessed using a NanoDrop 2000 spectrophotometer (Thermo Fisher Scientific, Waltham, MA, USA). Samples were considered acceptable when the A260/A280 ratio ranged between 1.8 and 2.0 and the concentration exceeded 50 ng/µL. Qualified DNA samples were stored at –20°C until further use.

### SNP discovery and sequence alignment

The target SNP loci C31799T (*GMPS*) and C34197G (*SRC*) were initially discovered and confirmed by bidirectional Sanger sequencing of PCR amplicons obtained from a pilot subset of 30 animals (15 bucks and 15 does). Obtained sequences were aligned against the goat reference genome (GenBank accession nos. NC_030808.1 and NC_030820.1) using DNAMAN software version 9.0. Only variants confirmed by both sequencing directions with a minimum Phred quality score of Q30 were retained.

### Primer design and PCR amplification

Gene-specific primers flanking the target intronic regions of *GMPS* and *SRC* were designed using Primer Premier 5.0 based on the goat reference genome (NC_030808.1 and NC_030820.1). All primer pairs were designed to have GC content of 45%–55% and were screened against potential primer-dimer formation. Primer sequences, optimal annealing temperatures (Tm), and expected amplicon sizes are presented in Table 1.

**Table 1:** Amplification and genotyping primers for target fragments of *Guanosine monophosphate synthase* (*GMPS*) and *Steroid receptor coactivator* (*SRC*).

Gene	Sense primer (5′→3′)	Anti-sense primer (5′→3′)	Tm (°C)	Fragment size (bp)	Genomic region
*GMPS*	ATCTGCTGTGGCATACTGA	CACTGATGAGCCTGTGAAA	48.4	754	111285624–111357129
*SRC*	TCATCTGTGCTTGACCCTA	CCCTAAGACCAAATAACCC	52	637	65852275–65906216

PCR was performed in a total volume of 50 µL containing:

- 25 µL of 2× SanTaq PCR Mix (Sangon Biotech, Shanghai, China)
- 1 µL DNA template (~50 ng)
- 2 µL of each primer (final concentration 0.4 µM each)
- nuclease-free water to final volume
  Thermal cycling conditions were as follows:
- Initial denaturation 94°C / 5 min
- 35 cycles of:
- 94°C / 30 s
- 48.4°C (*GMPS*) or 52 °C (*SRC*) / 30 s
- 72°C / 30 s
- Final extension 72°C / 10 min

### PCR product verification and Sanger sequencing

Amplification products were separated on 1.5% (w/v) agarose gels prepared in 1× TAE buffer and stained with GelRed nucleic acid stain. Electrophoresis was conducted at 130 V (constant power 180 W) for 20 min. A 100 bp DNA ladder (Thermo Fisher Scientific) was included for size determination. Amplicons showing a single band of the expected size were purified using the SanPrep Column PCR Product Purification Kit (Sangon Biotech, Shanghai, China) according to the manufacturer’s instructions. Purified products were submitted for bidirectional Sanger sequencing (Sangon Biotech Co., Ltd., Shanghai, China).

### Genotyping and quality control

Genotypes were manually called from bidirectional sequencing chromatograms using Chromas software version 2.6.6. A heterozygous call was accepted only when the secondary peak height reached at least 35% of the primary peak height. All chromatograms were independently scored by two researchers. Samples exhibiting poor sequence quality or a genotype call missing rate >5% were excluded. To assess repeatability, the complete workflow (PCR → sequencing → genotyping) was repeated for 10% of randomly selected samples, resulting in 100% genotype concordance.

### Haplotype inference

Haplotype phase was inferred using the SHEsis online platform with default parameters. To allow detection of sex-specific haplotype effects, haplotype reconstruction was performed separately for bucks and does. Rare haplotype combinations (population frequency <1%) were collapsed into a single “other” category for subsequent association analyses.

### Statistical analysis

#### Data distribution assumptions

Normality of continuous trait distributions was confirmed using the Shapiro–Wilk test (p > 0.05). Homogeneity of variance across genotype groups was verified with Levene’s test (p > 0.05). Both assumptions were satisfied, permitting the use of parametric statistical procedures.

#### Genotype–trait association analysis

Associations between genotypes and phenotypic traits were initially evaluated by one-way analysis of variance (ANOVA). Comprehensive analyses were performed using the following general linear mixed model:

Yijkl = μ + gj + bk + sl + eijkl

where Yijkl = observed phenotypic value μ = overall mean gj = fixed effect of genotype bk = fixed effect of age class sl = fixed effect of sex eijkl = random residual error

#### Multiple comparisons

When ANOVA indicated significant effects, pairwise comparisons were conducted using Duncan’s multiple range test. Results are presented as mean ± standard error of the mean (SEM). Levels of significance: p > 0.05 not significant; p < 0.05 significant (different lowercase letters); p < 0.01 highly significant (different uppercase letters).

#### Correlation, path, and regression analyses

Pearson correlation coefficients were calculated for pairwise trait relationships. Path analysis was performed to partition direct and indirect effects. Stepwise multiple regression was conducted (entry criterion p < 0.05; removal criterion p > 0.10). Multicollinearity was assessed using the variance inflation factor (VIF); all final model predictors had VIF < 3.

#### Software and significance threshold

Statistical analyses were performed using SPSS version 27.0 (ANOVA, correlation, regression), Microsoft Excel (genetic parameter calculations), SHEsis (haplotype inference), Chromas v. 2.6.6 (chromatogram viewing), and DNAMAN v. 9.0 (sequence alignment). The significance threshold was set at p < 0.05; p < 0.01 was considered highly significant. All continuous data are reported as mean ± SEM.

## RESULTS

### PCR amplification of *GMPS* and *SRC*

**Figure 1:**
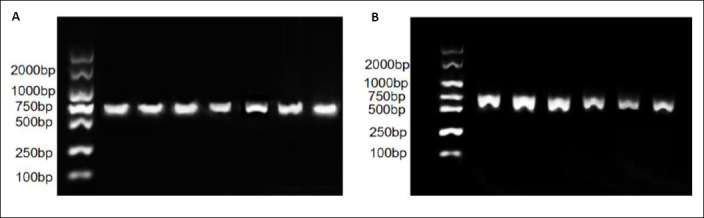
Polymerase chain reaction amplification of (A) *Guanosine monophosphate synthase* and (B) *Steroid receptor coactivator*.

Figure 1 shows the agarose gel electrophoresis results of PCR products for the target loci. (Left panel) A single specific band of 754 bp corresponding to the *GMPS* amplicon. (Right panel) A single specific band of 637 bp corresponding to the *SRC* amplicon. Successful and specific amplification suitable for downstream Sanger sequencing was confirmed by comparison with a 100 bp DNA ladder.

### Identification of SNP

**Figure 2:**
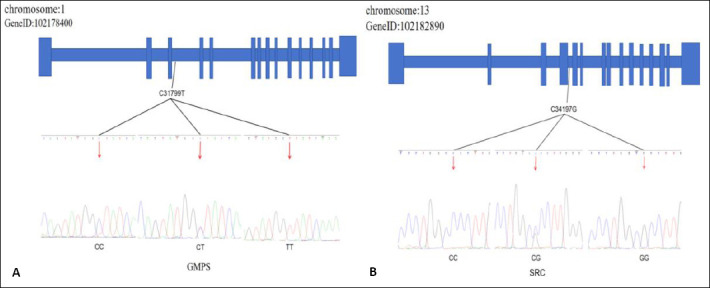
Identification of SNPs in (A) *Guanosine monophosphate synthase* and (B) *Steroid receptor coactivator* genes.

Figure 2 presents representative bidirectional sequencing chromatograms of the target polymorphic sites. (Left) *GMPS* C31799T locus showing homozygous CC, heterozygous CT, and homozygous TT genotypes. (Right) *SRC* C34197G locus showing homozygous CC, heterozygous CG, and homozygous GG genotypes. Arrows indicate the polymorphic nucleotide positions; clear double peaks in heterozygous samples confirm reliable detection of heterozygosity.

### Genetic polymorphisms of the *GMPS* and *SRC* genes in the LCG

Genotype frequencies, allele frequencies, polymorphism information content (PIC), expected heterozygosity (He), effective number of alleles (Ne), and Hardy–Weinberg equilibrium χ² test results at the two loci are presented in Table 2. The T allele at *GMPS* C31799T was predominant in both sexes (frequency 0.81 in bucks, 0.78 in does). The C allele at *SRC* C34197G was predominant (frequency 0.80 in bucks, 0.81 in does). PIC values ranged from 0.26 to 0.28, indicating moderate polymorphism at both loci. Significant deviation from the Hardy–Weinberg equilibrium was observed in most sex–locus combinations (p < 0.01).

**Table 2:** The genetic structure of the population at the polymorphic sites of the *Guanosine monophosphate synthase* (*GMPS*) and *Steroid receptor coactivator* (*SRC*) genes.

Gene	Loci	Gender	Genotype frequency	Allele frequency	PIC	He	Ne	χ²	p-value
*GMPS*	C31799T	Buck	CC 0.07, CT 0.23, TT 0.70	T 0.81, C 0.19	0.26	0.30	1.43	7.51	0.01
		Doe	CC 0.03, CT 0.38, TT 0.59	T 0.78, C 0.22	0.28	0.34	1.52	12.44	0.0004
*SRC*	C34197G	Buck	CC 0.59, CG 0.41, GG 0.00	C 0.80, G 0.20	0.27	0.33	1.48	7.27	0.007
		Doe	CC 0.69, CG 0.24, GG 0.07	C 0.81, G 0.19	0.26	0.31	1.45	54.25	1.76×10⁻¹³

### Effect of gene substitution

Additive and dominance effects estimated for the two loci are summarized in Table 3. Substitution of the C allele by the T allele at *GMPS* C31799T produced positive effects on production performance (average substitution effect 45.33% in bucks, 233.72% in does). Substitution of the C allele by the G allele at *SRC* C34197G produced negative effects (average substitution effect –25.11% in bucks, –386.07% in does).

**Table 3:** Gene substitution effect analysis of single-nucleotide polymorphisms in *Guanosine monophosphate synthase* (*GMPS*) and *Steroid receptor coactivator* (*SRC*) genes.

Gene	Loci	Gender	Dominant effect	Additive effect	B gene average effect	A gene average effect	Average effect of B instead of A
*GMPS*	C31799T	Buck	–18.00	34.00	8.40	–36.94	45.33
		Doe	70.00	273.00	51.28	–182.44	233.72
*SRC*	C34197G	Buck	12.50	–32.50	–19.98	5.14	–25.11
		Doe	–140.00	–300.00	–311.70	74.37	–386.07

### Effects of *GMPS* and *SRC* genes on cashmere production traits in LCG

Genotype means ± standard error and significance levels for cashmere production traits are presented in Table 4. In bucks, the *GMPS* TT genotype was associated with significantly longer staple length compared with CC (p < 0.01, partial η² = 0.096). In does, the *GMPS* TT genotype conferred superior CF (p < 0.01, partial η² = 0.025) and longer staple length (p < 0.01, partial η² = 0.022) compared with CC. The *SRC* CG genotype in does was associated with significantly finer cashmere fibers compared with CC (p < 0.01, partial η² = 0.045).

**Table 4:** Cashmere production performance of the *Guanosine monophosphate synthase* (*GMPS*) and *Steroid receptor coactivator* (*SRC*) genes (mean ± standard error).

Gender	Gene	Loci	Genotype (n/total)	Shearing quantity (g)	Fineness (μm)	Length (cm)	Coefficient of length variation (%)	Curl number	Short fiber rate (%)	Cashmere yield rate (%)
Buck	*GMPS*	C31799T	CC (6/78)	1850.00 ± 67.08	16.75 ± 0.37	118.80 ± 16.10^Aa^	46.60 ± 1.96	9.05 ± 0.16	0.09 ± 0.02	0.73 ± 0.01
			CT (18/78)	2066.67 ± 75.30	16.94 ± 0.14	95.47 ± 4.91^ABb^	49.95 ± 1.93	6.88 ± 0.77	0.13 ± 0.02	0.69 ± 0.02
			TT (54/78)	1944.44 ± 51.03	16.37 ± 0.17	90.76 ± 2.97^Bb^	57.31 ± 1.93	7.15 ± 0.54	0.21 ± 0.02	0.78 ± 0.02
Doe	*GMPS*	C31799T	CC (14/1022)	2350.00 ± 12.65^Aa^	18.09 ± 12.68^Aa^	61.60 ± 1.34^Bb^	25.39 ± 12.68^Bb^	5.24 ± 12.69	0.21 ± 12.68	0.54 ± 12.68^Bb^
			CT (448/1022)	1753.13 ± 12.71^Bb^	16.72 ± 12.71^Bb^	100.6 ± 1.53^Aa^	53.57 ± 12.71^Aa^	3.13 ± 12.71	0.17 ± 12.71	0.74 ± 12.71^Aa^
			TT (560/1022)	1798.75 ± 12.62^Bb^	16.52 ± 12.62^Bb^	97.73 ± 1.26^Aa^	54.77 ± 12.62^Aa^	8.75 ± 12.62	0.16 ± 12.62	0.76 ± 12.62^Aa^
Buck	*SRC*	C34197G	CC (44/80)	2000.00 ± 36.00	16.84 ± 0.15	91.35 ± 2.61	54.96 ± 1.96	8.02 ± 0.37	0.13 ± 0.02^ABab^	0.70 ± 0.02
			CG (36/80)	2127.78 ± 52.24	16.58 ± 0.18	98.36 ± 4.70	56.77 ± 1.86	6.98 ± 0.58	0.27 ± 0.03^Aa^	0.79 ± 0.03
Doe	*SRC*	C34197G	CC (686/1022)	1828.57 ± 12.01^Aa^	16.81 ± 0.05^Aa^	96.77 ± 1.21	53.85 ± 0.54	6.43 ± 0.15^Bb^	0.18 ± 0.01^Aa^	0.73 ± 0.01^Bb^
			CG (252/1022)	1734.86 ± 15.04^Bb^	16.18 ± 0.07^Bb^	97.79 ± 1.74	52.34 ± 0.67	7.58 ± 0.24^Aa^	0.12 ± 0.01^Ba^	0.79 ± 0.01^Aa^
			GG (84/1022)	1816.67 ± 38.51^ABa^	16.42 ± 0.14^Bb^	99.07 ± 3.45	53.79 ± 1.58	5.70 ± 0.45^Bb^	0.20 ± 0.01^Aa^	0.78 ± 0.01^Aa^

Different lowercase letters indicate significant differences (p < 0.05); different uppercase letters indicate highly significant differences (p < 0.01). Sample sizes were limited for some genotype groups (e.g., *GMPS* CC in bucks n = 6, *GMPS* CC in does n = 14).

Effects of *GMPS* and *SRC* on body size traits in LCG Genotype means ± standard error for body conformation traits are shown in Table 5. The *GMPS* CC genotype was associated with superior body size traits in both sexes. In bucks, CC conferred significantly greater height at the sacrum and waist height (p < 0.01, partial η² = 0.319 and 0.216, respectively). In does, CC was linked to significantly greater sacrum height, chest depth, chest width, and waist width (p < 0.01, partial η² = 0.009–0.020). The *SRC* CG genotype in does was associated with significantly greater body height, chest depth, and waist height compared with CC and GG (p < 0.05, partial η² = 0.012–0.067).

**Table 5:** Analysis of the body size traits of the *Guanosine monophosphate synthase* (*GMPS*) and *Steroid receptor coactivator* (*SRC*) genes in Liaoning cashmere goat (mean ± standard error).

Gender	Gene	Loci	Genotype (n/total)	Body height (cm)	Height at the sacrum (cm)	Body length (cm)	Chest depth (cm)	Chest width (cm)	Waist width (cm)	Chest circumference (cm)	Tube circumference (cm)	Waist height (cm)
Doe	*GMPS*	C31799T	CC (32/1064)	64.25 ± 0.45	67.50 ± 0.53^Aa^	71.75 ± 1.11^Bb^	33.80 ± 0.27^Aa^	25.00 ± 0.22^Aa^	25.88 ± 0.49^Aa^	100.25 ± 1.31^ABab^	10.25 ± 0.19^Aa^	65.00 ± 0.77
			CT (400/1064)	63.69 ± 0.19	65.09 ± 0.19^Bb^	77.65 ± 0.33^Aa^	32.31 ± 0.14^Bb^	23.24 ± 0.22^Bb^	22.52 ± 0.23^Bb^	97.87 ± 0.40^Bb^	9.48 ± 0.05^Bb^	63.97 ± 0.22
			TT (632/1064)	64.06 ± 0.13	65.56 ± 0.13^Bb^	79.10 ± 0.24^Aa^	32.52 ± 0.11^Bb^	23.99 ± 0.15^ABab^	23.48 ± 0.18^Bb^	101.71 ± 0.40^Aa^	9.37 ± 0.04^Bb^	64.56 ± 0.19
Buck	*GMPS*	C31799T	CC (6/81)	75.50 ± 0.19	80.25 ± 1.04^Aa^	89.50 ± 0.57	34.75 ± 0.28	25.25 ± 1.04^ab^	19.00 ± 1.51	104.75 ± 1.80^ABab^	12.50 ± 0.23	76.00 ± 0.38^Aa^
			CT (18/81)	75.50 ± 0.82	72.42 ± 0.54^Bb^	86.83 ± 1.42	36.58 ± 0.30	23.50 ± 0.76^b^	19.08 ± 0.58	101.58 ± 0.80^Bb^	12.75 ± 0.33	69.58 ± 0.97^Bb^
			TT (57/81)	74.99 ± 0.36	72.45 ± 0.36^Bb^	84.11 ± 1.65	33.96 ± 0.80	28.02 ± 0.63^a^	21.53 ± 0.34	106.68 ± 0.47^Aa^	12.68 ± 0.11	68.66 ± 0.40^Bb^
Doe	*SRC*	C34197G	CC (693/1035)	63.77 ± 0.14^Ab^	65.77 ± 0.13^Aa^	79.44 ± 0.23^Aa^	32.15 ± 0.11^b^	23.32 ± 0.15^ab^	22.84 ± 0.17^Aa^	99.54 ± 0.36^Aa^	9.47 ± 0.04	63.68 ± 0.17^Bb^
			CG (261/1035)	64.66 ± 0.21^Aa^	66.09 ± 0.18 ^Aa^	78.19 ± 0.36^Aa^	32.86 ± 0.18^a^	24.06 ± 0.28^a^	22.99 ± 0.30^Aa^	100.77 ± 0.67^Aa^	9.52 ± 0.06	65.14 ± 0.28^Aa^
			GG (81/1035)	62.17 ± 0.35^Bc^	63.39 ± 0.36 ^Bb^	75.33 ± 0.80^Bb^	32.38 ± 0.20^ab^	23.06 ± 0.45^b^	21.09 ± 0.47^Bb^	94.86 ± 0.70^Bb^	9.49 ± 0.14	60.28 ± 0.35^Cc^
Buck	*SRC*	C34197G	CC (48/84)	75.46 ± 0.43	73.25 ± 0.36	81.79 ± 2.28	32.33 ± 1.04^b^	27.92 ± 0.86	20.48 ± 0.42	104.98 ± 0.72	12.71 ± 0.18	69.50 ± 0.56
			CG (36/84)	74.94 ± 0.37	73.33 ± 0.69	87.39 ± 0.60	37.11 ± 0.24^a^	25.81 ± 0.54	21.17 ± 0.42	105.10 ± 0.60	12.56 ± 0.11	69.50 ± 0.48

Different lowercase letters indicate significant differences (p < 0.05); different uppercase letters indicate highly significant differences (p < 0.01). Sample sizes were limited for some genotype groups (e.g., *GMPS* CC in bucks n = 6).

Different lowercase letters indicate significant differences (p < 0.05); different uppercase letters indicate highly significant differences (p < 0.01). Sample sizes were limited for some genotype groups (e.g., *GMPS* CC in bucks n = 6).

### Effects of *GMPS* and *SRC* genotypes on slaughter performance in LCG

Genotype means ± standard error for slaughter performance traits are presented in Table 6. In bucks, the *GMPS* TT genotype was superior for live weight before slaughter, net meat percentage of carcass, and back fat thickness (p < 0.01, partial η² = 0.151–0.305). In does, TT was associated with higher slaughter rate and GR value (p < 0.01, partial η² = 0.542–0.947). For *SRC* in does, the CC genotype showed advantages in live weight before slaughter, carcass weight, net meat weight, and GR value (p < 0.01, partial η² = 0.225–0.419).

**Table 6:** Liaoning cashmere goat slaughter performance: associations with *Guanosine monophosphate synthase* (*GMPS*) and *Steroid receptor coactivator* (*SRC*) genes (mean ± standard error).

Gender	Gene	Loci	Genotype (n/total)	Live weight before slaughter (kg)	Carcass weight (kg)	Net meat weight (kg)	Slaughter rate (%)	Net meat consumption rate (%)	Net carcass meat percentage (%)	Eye muscle area (cm²)	GR value (mm)	Back fat thickness (mm)
Buck	*GMPS*	C31799T	CC (3/39)	46.80 ± 0.50^ab^	22.00 ± 0.61	17.40 ± 0.21	47.01 ± 0.30^Bb^	37.18 ± 0.45	79.09 ± 0.31^Aa^	19.65 ± 0.23	4.28 ± 0.15^Bb^	1.06 ± 0.25^b^
			CT (6/39)	41.55 ± 0.25^b^	21.75 ± 0.16	16.25 ± 0.11	52.34 ± 0.07^Aa^	39.11 ± 0.04	74.71 ± 0.02^Bb^	20.88 ± 1.11	7.94 ± 0.10^Aa^	1.63 ± 0.21^ab^
			TT (30/39)	47.87 ± 0.94^a^	24.81 ± 0.65	19.63 ± 0.60	51.72 ± 0.64^ABa^	40.84 ± 0.67	78.86 ± 0.48^Aa^	22.70 ± 0.88	6.21 ± 0.38^Aab^	2.25 ± 0.18^a^
Doe	*GMPS*	C31799T	CT (25/60)	46.78 ± 1.20	22.78 ± 0.45	18.78 ± 0.37	48.91 ± 0.32^b^	40.33 ± 0.33	82.44 ± 0.29	18.94 ± 0.46	6.98 ± 0.15^b^	2.47 ± 0.09
			TT (35/60)	45.67 ± 0.92	23.39 ± 0.56	19.17 ± 0.53	51.13 ± 0.38^a^	41.84 ± 0.47	81.73 ± 0.36	19.88 ± 0.39	8.78 ± 0.30^a^	3.11 ± 0.16
Buck	*SRC*	C34197G	CC (30/60)	49.35 ± 1.06^Aa^	24.68 ± 0.60^Aa^	20.33 ± 0.56^Aa^	50.01 ± 0.47	41.14 ± 0.52	82.20 ± 0.27	19.59 ± 0.22^Aa^	9.16 ± 0.25^Aa^	3.04 ± 0.20
			CG (20/60)	44.00 ± 0.87^Bb^	22.20 ± 0.30^ABb^	18.10 ± 0.34^ABb^	50.63 ± 0.48	41.25 ± 0.55	81.43 ± 0.55	20.85 ± 0.61^Aa^	7.13 ± 0.30^Bb^	2.72 ± 0.07
			GG (10/60)	40.75 ± 0.80^Bb^	20.35 ± 0.35^Bb^	16.85 ± 0.38^Bb^	49.97 ± 0.13	41.32 ± 0.11	82.72 ± 0.44	16.43 ± 0.57^Bb^	6.43 ± 0.16^Bb^	2.50 ± 0.06
Doe	*SRC*	C34197G	CC (24/32)	47.22 ± 1.35	24.15 ± 0.92	18.97 ± 0.90	50.92 ± 0.76	39.78 ± 0.89	77.97 ± 0.80	20.02 ± 0.46^b^	6.26 ± 0.48	2.31 ± 0.26
			CG (8/32)	49.60 ± 1.28	26.05 ± 0.35	20.45 ± 0.36	52.64 ± 0.64	41.29 ± 0.35	78.47 ± 0.30	24.02 ± 1.66^a^	7.16 ± 0.57	1.79 ± 0.04

Different lowercase letters indicate significant differences (p < 0.05); different uppercase letters indicate highly significant differences (p < 0.01). Sample sizes were limited for some genotype groups (e.g., *GMPS* CC in bucks n = 3, *SRC* GG in bucks n = 10).

Different lowercase letters indicate significant differences (p < 0.05); different uppercase letters indicate highly significant differences (p < 0.01). Sample sizes were limited for some genotype groups (e.g., *GMPS* CC in bucks n = 3, *SRC* GG in bucks n = 10).

### Effects of *GMPS* and *SRC* gene variations on meat quality traits in LCG

Genotype means ± standard error for meat quality parameters are presented in Table 7. In bucks, the *GMPS* CC genotype was associated with superior meat color a* value and dry matter content (p < 0.01, partial η² = 0.296 and 0.499, respectively). The *SRC* CG genotype in bucks showed more favorable meat color L* and b* values and higher protein content compared with CC (p < 0.01, partial η² = 0.772–0.991). In does, the *SRC* CC genotype demonstrated advantages in protein content, fat content, and shear force compared with CG (p < 0.01, partial η² = 0.259–0.420).

**Table 7:** Analysis of the meat quality traits of the *Guanosine monophosphate synthase* (*GMPS*) and *Steroid receptor coactivator* (*SRC*) genes in Liaoning cashmere goat (mean ± standard error).

Gender	Gene	Loci	Genotype (n/total)	Meat color (L)	Meat color (a)	Meat color (b)	pH	Dry matter content (%)	Protein content (%)	Fat content (%)	Drip loss (%)	Cooked meat (%)	Shear force (N)
Buck	*GMPS*	C31799T	CC (3/39)	28.45 ± 0.12	18.54 ± 0.20^Aa^	1.51 ± 0.04	6.02 ± 0.08	28.60 ± 0.25^Aa^	21.00 ± 0.15	1.50 ± 0.07	1.12 ± 0.01^Cc^	70.86 ± 0.09^a^	70.85 ± 0.00^Bb^
			CT (6/39)	28.26 ± 0.21	13.06 ± 0.10^Bc^	1.68 ± 0.02	6.04 ± 0.03	25.35 ± 0.11^Bb^	21.81 ± 0.10	2.31 ± 0.08	1.52 ± 0.01^Bb^	66.30 ± 0.04^b^	82.34 ± 0.26^Aa^
			TT (30/39)	29.89 ± 0.18	14.72 ± 0.16^Bb^	1.74 ± 0.04	6.10 ± 0.02	25.76 ± 0.15^Bb^	21.38 ± 0.12	1.83 ± 0.09	1.73 ± 0.01^Aa^	65.27 ± 0.43^b^	74.66 ± 0.79^ABab^
Doe	*GMPS*	C31799T	CT (25/60)	30.33 ± 0.12	13.23 ± 0.03^Bb^	1.87 ± 0.03	5.91 ± 0.01	29.48 ± 0.21	20.67 ± 0.07	2.29 ± 0.03	1.77 ± 0.01	69.65 ± 0.12	70.19 ± 0.59
			TT (35/60)	29.56 ± 0.07	15.05 ± 0.05^Aa^	1.81 ± 0.02	6.00 ± 0.01	26.73 ± 0.05	21.00 ± 0.04	2.34 ± 0.05	1.71 ± 0.01	67.65 ± 0.20	71.56 ± 0.24
Buck	*SRC*	C34197G	CC (24/32)	28.15 ± 0.17^Bb^	14.66 ± 0.44	1.62 ± 0.02^Bb^	6.11 ± 0.03^b^	26.43 ± 0.35	20.86 ± 0.21^Bb^	1.96 ± 0.08^Aa^	1.58 ± 0.05	65.64 ± 1.03	76.26 ± 1.41^a^
			CG (8/32)	31.22 ± 0.02^Aa^	14.02 ± 0.16	2.21 ± 0.22^Aa^	6.25 ± 0.02^a^	25.03 ± 0.00	22.50 ± 0.15^Aa^	1.11 ± 0.08^Bb^	1.79 ± 0.01	67.68 ± 0.99	69.40 ± 1.13^b^
Doe	*SRC*	C34197G	CC (30/60)	28.69 ± 0.36^Bc^	14.39 ± 0.23^Aa^	1.80 ± 0.08	5.97 ± 0.03	26.97 ± 0.22^Bb^	21.47 ± 0.20^Aa^	2.66 ± 0.21^Aa^	1.67 ± 0.06	69.65 ± 0.71	75.68 ± 1.30^Aa^
			CG (20/60)	31.62 ± 0.36^Aa^	14.80 ± 0.37^Aa^	1.77 ± 0.15	5.96 ± 0.02	30.15 ± 1.11^Aa^	19.79 ± 0.20^Bb^	1.61 ± 0.10^Bb^	1.80 ± 0.05	67.37 ± 1.24	63.99 ± 2.13^Bb^
			GG (10/60)	29.98 ± 0.47^ABb^	12.97 ± 0.12^Bb^	2.07 ± 0.22	5.96 ± 0.01	26.04 ± 0.18^Bb^	21.19 ± 0.06^Aa^	2.70 ± 0.13^Aa^	1.81 ± 0.01	67.23 ± 0.44	70.92 ± 1.90^ABa^

Different lowercase letters indicate significant differences (p < 0.05); different uppercase letters indicate highly significant differences (p < 0.01). Sample sizes were limited for some genotype groups (e.g., *GMPS* CC in bucks n = 3, *SRC* CG in bucks n = 8, *SRC* GG in does n = 10).

### Effects of *GMPS* and *SRC* gene variations on milk production traits in LCG

Milk composition parameters by genotype are shown in Table 8. For *GMPS* in does, the CT and TT genotypes exhibited superior performance compared with CC for fat, crude protein, urea nitrogen, solids-not-fat, and total solids (p < 0.05, partial η² = 0.010–0.029). For *SRC*, the CC and CG genotypes demonstrated better performance than GG for fat, urea nitrogen, solids-not-fat, and total solids (p < 0.01, partial η² = 0.040–0.064).

**Table 8:** Association between *Guanosine monophosphate synthase* (*GMPS*) and *Steroid receptor coactivator* (*SRC*) genes and Liaoning cashmere goat milk performance (mean ± standard error).

Name	Gene	Loci	Genotype (n/total)	Fat	Crude protein	Lactose	Urea N	SnF	TS	Conductivity	Health index
Doe	*GMPS*	C31799T	CC (18/354)	5.98 ± 0.30^b^	4.43 ± 0.03^b^	5.34 ± 0.02	38.63 ± 0.49^b^	18.04 ± 0.23^b^	10.23 ± 0.03 b	16.48 ± 0.29^Bb^	766.23 ± 6.45
			CT (108/354)	7.11 ± 0.27^a^	5.78 ± 0.36^a^	5.14 ± 0.06	42.29 ± 1.09^a^	19.74 ± 0.51^a^	11.60 ± 0.35^a^	19.10 ± 0.38^Aa^	749.43 ± 6.36
			TT (228/354)	7.14 ± 0.17^a^	5.16 ± 0.12^ab^	5.11 ± 0.05	39.92 ± 0.37^ab^	18.62 ± 0.17^ab^	10.85 ± 0.11^ab^	18.38 ± 0.20^Aa^	775.88 ± 7.52
Doe	*SRC*	C34197G	CC (216/328)	6.80 ± 0.16^Aa^	5.14 ± 0.12^ab^	5.14 ± 0.03^Ab^	40.20 ± 0.40^Aa^	18.76 ± 0.18^Aa^	10.86 ± 0.11	18.03 ± 0.19^Aa^	775.57 ± 4.21^Bb^
			CG (88/328)	7.60 ± 0.27^Aa^	5.69 ± 0.28^a^	4.74 ± 0.11^Bc^	40.92 ± 0.39^Aa^	19.10 ± 0.18^Aa^	11.05 ± 0.23	19.09 ± 0.40^Aa^	841.96 ± 18.89^Aa^
			GG (24/328)	5.05 ± 0.30^Bb^	4.77 ± 0.05^b^	5.46 ± 0.04^Aa^	36.57 ± 0.44^Bb^	17.07 ± 0.21^Bb^	10.76 ± 0.05	16.05 ± 0.32^Bb^	752.43 ± 4.61^Bb^

Different lowercase letters indicate significant differences (p < 0.05); different uppercase letters indicate highly significant differences (p < 0.01). SnF = solids-not-fat; TS = total solids. Sample size was limited for *GMPS* CC in does (n = 18).

### Effects of *GMPS* and *SRC* gene variants on lambing traits in LCG

Litter size means by genotype are presented in Table 9. No significant differences in the number of lambs per doe were detected across genotypes at either the *GMPS* C31799T or *SRC* C34197G loci.

**Table 9:** Effects of *Guanosine monophosphate synthase* (*GMPS*) and *Steroid receptor coactivator* (*SRC*) gene variants on Liaoning cashmere goat lambing traits (mean ± standard error).

Name	Gene	Loci	Genotype (n/total)	Number of lambs
Doe	*GMPS*	C31799T	CT (145/325)	1.38 ± 0.04
			TT (180/325)	1.28 ± 0.03
			CC (215/325)	1.35 ± 0.03
Doe	*SRC*	C34197G	CG (85/325)	1.29 ± 0.05
			GG (25/325)	1.40 ± 0.10
			CT (145/325)	1.38 ± 0.04

### Relationships between fiber fineness and production traits in Liaoning cashmere goat

Pearson correlation coefficients between CF and other fiber and production traits are shown in Table 10. Fiber fineness exhibited highly significant positive correlations with shearing quantity (bucks r = 0.41, does r = 0.38; p < 0.01) and highly significant negative correlations with cashmere yield rate (bucks r = –0.90, does r = –0.93; p < 0.01) in both sexes.

**Table 10:** Relationships between fiber fineness and Liaoning cashmere goat production traits.

Trait	Buck fineness (μm)	Doe fineness (μm)	Shearing quantity (g) Buck	Shearing quantity (g) Doe	Length (cm) Buck	Length (cm) Doe	Coefficient of variation in length Buck	Coefficient of variation in length Doe	Curl number Buck	Curl number Doe	Short fiber rate (%) Buck	Short fiber rate (%) Doe	Cashmere yield rate (%) Buck	Cashmere yield rate (%) Doe
Fineness (μm)	–	–	0.41**	0.38**	0.09	–	–0.06	–0.32**	–0.09	–0.14**	–0.26**	–0.15**	–0.90**	–0.93**
Shearing quantity (g)	0.41**	0.38**	–	–	–0.19	0.12**	0.25*	–0.32**	0.12	–0.18**	0.03	–0.06*	–0.21*	–0.39**
Length (cm)	0.09	–	–0.19	0.12**	–	–	0.07	–0.47**	0.38**	0.04	–0.26**	–0.37**	0.00	–0.35**
Coefficient of variation in length	–0.06	–0.32**	0.25*	–0.32**	0.07	–0.47**	–	–	0.77**	0.61**	0.25*	0.39**	0.25*	0.39**
Curl number	–0.09	–0.14**	0.12	–0.18**	0.38**	0.04	0.77**	0.61**	–	–	–0.25*	–0.15**	0.21*	0.12**
Short fiber rate (%)	–0.26**	–0.15**	0.03	–0.06*	–0.26**	–0.37**	0.25*	0.39**	–0.25*	–0.15**	–	–	0.38**	0.18**
Cashmere yield rate (%)	–0.90**	–0.93**	–0.21*	–0.39**	0.00	–0.35**	0.25*	0.39**	0.21*	0.12**	0.38**	0.18**	–	–

### Path analysis of CF on production traits

Direct and indirect path coefficients from production traits to CF are shown in Table 11. In does, the strongest direct negative effect was exerted by cashmere yield rate (–0.92), followed by coefficient of variation of length (0.10). In bucks, cashmere yield rate (–0.89) and shearing quantity (0.23) were the dominant direct contributors.

**Table 11:** Path analysis of cashmere fineness on Liaoning cashmere goat production traits.

Gender	Independent variable	Correlation coefficient	Direct path coefficient	Indirect effect via Shearing quantity (g)	Indirect effect via Length (cm)	Indirect effect via Coefficient of variation in length	Indirect effect via Curl number	Indirect effect via Short fiber rate (%)	Indirect effect via Cashmere yield rate (%)
Doe	Shearing quantity (g)	0.38	0.05	–	0.01	–0.01	–0.01	0.00	–0.02
	Length (cm)	0.34	0.06	0.01	–	–0.03	0.00	–0.02	–0.02
	Coefficient of variation in length	–0.32	0.10	–0.03	–0.05	–	0.01	0.06	0.04
	Curl number	–0.14	–0.03	0.01	0.00	0.00	–	0.01	0.00
	Short fiber rate (%)	–0.15	–0.02	0.00	0.01	–0.01	0.00	–	0.00
	Cashmere yield rate (%)	–0.93	–0.92	0.36	0.32	–0.36	–0.11	–0.17	–
Buck	Shearing quantity (g)	0.41	0.23	–	–	–	–	–0.04	0.06
	Short fiber rate (%)	–0.26	0.06	–0.01	–	–	–	–	0.00
	Cashmere yield rate (%)	–0.90	–0.89	–0.22	–	–	–	–0.06	–

### Stepwise regression analysis of cashmere traits and fineness

Multiple linear regression models with CF as the dependent variable are presented in Table 12. For bucks: CF = 0.001 × shearing quantity – 0.07 × cashmere yield rate + 20.553 (R² = 0.85). For does: CF = 0.0001 × shearing quantity – 0.079 × cashmere yield rate + 22.307 (R² = 0.86). Both models explained more than 85% of the variation in CF.

**Table 12:** Stepwise regression analysis of cashmere traits and fineness in Liaoning cashmere goat.

Gender	Model	R²	Adjusted R²	Standard error of the estimate	F-value	p-value
Buck	CF = 22.360 – 0.07 CY	0.80	0.80	0.51	306.39	0.00
	CF = 0.001 SQ – 0.07 CY + 20.553	0.85	0.85	0.44	218.42	0.00
Doe	CF = 22.450 – 0.078 CY	0.86	0.86	0.47	6356.68	0.00
	CF = 0.0001 SQ – 0.079 CY + 22.307	0.86	0.86	0.47	3231.52	0.00

### Association of CF with slaughter traits in LCG

Pearson correlation coefficients between CF and slaughter traits are shown in Table 13. Fineness exhibited highly significant negative correlations with several carcass merit indicators, including slaughter rate, net meat consumption rate, and net carcass meat percentage in both sexes (p < 0.01).

**Table 13:** Association between cashmere fineness and slaughter traits in Liaoning cashmere goat.

Trait	Buck fineness (μm)	Doe fineness (μm)	Live weight before slaughter (kg) Buck	Live weight before slaughter (kg) Doe	Carcass weight (kg) Buck	Carcass weight (kg) Doe	Net meat weight (kg) Buck	Net meat weight (kg) Doe	Slaughter rate (%) Buck	Slaughter rate (%) Doe	Net meat consumption rate (%) Buck	Net meat consumption rate (%) Doe	Net carcass meat percentage (%) Buck	Net carcass meat percentage (%) Doe	Eye muscle area (cm²) Buck	Eye muscle area (cm²) Doe	GR value (mm) Buck	GR value (mm) Doe	Back fat thickness (mm) Buck	Back fat thickness (mm) Doe
Fineness (μm)	–	–	0.11	–0.20	–0.08	–0.33*	–0.13	–0.39*	–0.477**	–0.48**	–0.48**	–0.54**	–0.32*	–0.57**	0.24	–0.48**	–0.18	–0.09	–0.31*	–0.28
Live weight before slaughter (kg)	0.11	–0.20	–	–	0.92**	0.90**	0.88**	0.90**	–0.197	0.21	–0.02	0.45**	0.31*	0.70**	0.31*	–0.19	0.32*	0.27	0.37**	0.61**
Carcass weight (kg)	–0.08	–0.33*	0.92**	0.90**	–	–	0.99**	0.99**	0.194	0.62**	0.35**	0.78**	0.51**	0.73**	0.20	0.07	0.34**	0.45**	0.59**	0.63**
Net meat weight (kg)	–0.13	–0.39*	0.88**	0.90**	0.99**	0.99**	–	–	0.269*	0.59**	0.45**	0.80**	0.62**	0.82**	0.13	0.05	0.30*	0.46**	0.61**	0.68**
Slaughter rate (%)	–0.477**	–0.48**	–0.197	0.21	0.194	0.62**	0.269*	0.59**	–	–	0.94**	0.919**	0.51**	0.381*	–0.27*	0.504**	–0.03	0.544**	0.52**	0.28
Net meat consumption rate (%)	–0.48**	–0.54**	–0.02	0.45**	0.35**	0.78**	0.45**	0.80**	0.94**	0.919**	–	–	0.76**	0.71**	–0.33*	0.38*	–0.05	0.59**	0.53**	0.51**
Net carcass meat percentage (%)	–0.32*	–0.57**	0.31*	0.70**	0.51**	0.73**	0.62**	0.82**	0.51**	0.381*	0.76**	0.71**	–	–	–0.33*	0.00	–0.07	0.36**	0.36**	0.67**
Eye muscle area (cm²)	0.24	–0.48**	0.31*	–0.19	0.20	0.07	0.13	0.05	–0.27*	0.504**	–0.33*	0.38*	–0.33*	0.00	–	–	0.15	–0.28	–0.01	–0.24
GR value (mm)	–0.18	–0.09	0.32*	0.27	0.34**	0.45**	0.30*	0.46**	–0.03	0.544**	–0.05	0.59**	–0.07	0.38*	0.15	–0.28	–	–	0.60**	0.32*
Back fat thickness (mm)	–0.31*	–0.28	0.37**	0.61**	0.59**	0.63**	0.61**	0.68**	0.52**	0.28	0.53**	0.51**	0.36**	0.67**	–0.01	–0.24	0.60**	0.32*	–	–

** p < 0.01;

* p < 0.05; no symbol = p > 0.05.

### Path analysis of slaughter traits on CF

Direct and indirect path coefficients from slaughter traits to CF are presented in Table 14. In bucks, net meat weight exerted the strongest direct positive effect on fineness (31.82). In does, the slaughter rate was the primary direct negative contributor (–1.05).

**Table 14:** Path analysis of Liaoning cashmere goat slaughter traits on cashmere fineness.

Gender	Independent variable	Correlation coefficient	Direct path coefficient	Indirect action (path coefficient) Net meat weight (kg)	Indirect action (path coefficient) Slaughter rate (%)	Indirect action (path coefficient) Net meat consumption rate (%)	Indirect action (path coefficient) Net carcass meat percentage (%)	Indirect action (path coefficient) Eye muscle area (cm²)	Indirect action (path coefficient) Back fat thickness (mm)
Buck	Net meat weight (kg)	–0.39	31.82	–	18.84	25.36	26.00	1.50	–
	Slaughter rate (%)	–0.40	10.52	6.23	–	9.67	4.01	5.30	–
	Net meat consumption rate (%)	–0.54	–29.92	–23.84	–27.49	–	–21.36	–11.28	–
	Net carcass meat percentage (%)	–0.57	5.89	4.81	2.24	4.21	–	0.02	–
	Eye muscle area (cm²)	–0.48	–0.10	0.00	–0.05	–0.04	0.00	–	–
Doe	Slaughter rate (%)	–0.48	–1.05	–	–	–	–0.53	–	–0.54
	Net carcass meat percentage (%)	–0.32	–0.52	–	–0.26	–	–	–	–0.19
	Back fat thickness (mm)	–0.31	0.04	–	0.02	–	0.01	–	–

### Stepwise regression model of slaughter traits for CF

Optimal multiple regression equations for CF based on slaughter traits are shown in Table 15. For does: CF = 27.558 – 0.217 × slaughter rate (R² = 0.23). For bucks: CF = 36.723 – 0.230 × net carcass meat percentage – 0.118 × eye muscle area (R² = 0.55).

**Table 15:** Stepwise regression model of slaughter traits for fineness of cashmere in Liaoning cashmere goat.

Gender	Model	R²	Adjusted R²	Standard error of the estimate	F-value	p-value
Buck	CF = 34.164 – 0.230 CP	0.33	0.31	0.92	18.02	0.00
	CF = 36.723 – 0.230 CP – 0.118 EM	0.55	0.53	0.77	22.08	0.00
Doe	CF = 27.558 – 0.217 DP	0.23	0.21	0.98	17.08	0.00

CF = Cashmere fineness; CP = Net carcass meat percentage; EM = Eye muscle area; DP = Slaughter rate.

### Association of *GMPS* and *SRC* haplotypes with production traits

Nine haplotype combinations were inferred from the two SNP loci using SHEsis software (Table 16). Bucks displayed six combinations: CCCC, CCCG, CTCC, CTCG, TTCC, TTCG. Does displayed seven combinations: CCGG, CTCC, CTCG, CTGG, TTCC, TTCG, TTGG.

**Table 16:** Haplotype combination analysis of the *Guanosine monophosphate synthase* (*GMPS*) and *Steroid receptor coactivator* (*SRC*) genes.

Haplotype combination	CC	CG	GG
CC	CCCC	CCCG	CCGG
CT	CTCC	CTCG	CTGG
TT	TTCC	TTCG	TTGG

### Haplotype effects on cashmere production performance

Haplotype means ± standard error for cashmere traits are presented in Table 17. In bucks, the CCCC combination was associated with the finest cashmere fibers. In does, the TTGG combination outperformed the other haplotypes in fineness, coefficient of variation of length, and curl number.

**Table 17:** Haplotype effects of *Guanosine monophosphate synthase* (*GMPS*) and *Steroid receptor coactivator* (*SRC*) genes on cashmere production performance in Liaoning cashmere goat (mean ± standard error).

Name	Haplotype	Shearing quantity (g)	Fineness (μm)	Length (cm)	Coefficient of variation in length (%)	Curl number	Short fiber rate (%)	Cashmere yield rate (%)
Buck	CCCC (4/80)	1700.00 ± 30.15^Cc^	15.92 ± 21.31^Bc^	82.80 ± 5.30^Cc^	50.98 ± 36.70^ab^	9.40 ± 29.40	0.09 ± 32.00^Bb^	0.75 ± 28.60^ab^
	CCCG (4/80)	2000.00 ± 15.05^BCb^	17.57 ± 50.10^Aa^	154.80 ± 3.06^Aa^	42.22 ± 46.80^b^	8.70 ± 52.60	0.09 ± 49.03^Bb^	0.71 ± 47.62 ab
	CTCC (16/80)	1925.00 ± 62.04^BCbc^	17.13 ± 62.04^ABab^	100.05 ± 3.03^BCbc^	52.57 ± 62.04^ab^	8.43 ± 62.04	0.08 ± 62.04^Bb^	0.68 ± 62.04^b^
	CTCG (4/80)	2500.00 ± 20.50^Aa^	17.25 ± 35.20^ABab^	113.80 ± 2.45^Bb^	55.99 ± 42.60^ab^	7.60 ± 50.70	0.17 ± 36.50^ABab^	0.67 ± 41.20^b^
	TTCC (24/80)	2100.00 ± 41.87^Bb^	16.80 ± 41.87^ABabc^	86.97 ± 3.42^Cc^	57.22 ± 41.87^a^	7.52 ± 41.87	0.17 ± 41.87^ABab^	0.71 ± 41.87^ab^
	TTCG (28/80)	2092.86 ± 62.08^Bb^	16.34 ± 62.08^ABbc^	88.09 ± 3.43^Cc^	58.96 ± 62.08^a^	6.64 ± 62.08	0.31 ± 62.08^Aa^	0.82 ± 62.08^a^
Doe	CCGG (15/1035)	2350.00 ± 15.20^Aa^	18.09 ± 0.20^Aa^	61.60 ± 2.80^Cc^	25.39 ± 0.90^Cd^	6.78 ± 0.30	0.21 ± 0.02^Aa^	0.54 ± 0.02^Cd^
	CTCC (270/1035)	1783.33 ± 17.34^Ccd^	16.87 ± 0.08^BCb^	100. ± 2.02^Bb^	52.80 ± 1.02^Bbc^	6.73 ± 0.23^ABb^	0.15 ± 0.01^Ab^	0.72 ± 0.01^Bc^
	CTCG (135/1035)	1700.00 ± 20.86^Cd^	16.14 ± 0.11^DEc^	97.9 ± 2.95^Bb^	53.66 ± 1.10^Bbc^	6.57 ± 0.42^ABb^	0.20 ± 0.01^Aab^	0.79 ± 0.01^Bb^
	CTGG (30/1035)	1950.00 ± 37.14^Bb^	16.92 ± 0.07^Bb^	135. ± 0.84^Aa^	56.82 ± 0.76^ABab^	4.65 ± 0.86^Bc^	0.21 ± 0.02^Aa^	0.75 ± 0.01^Bbc^
	TTCC (405/1035)	1838.89 ± 15.78^BCbc^	16.71 ± 0.06^BCDb^	96.89 ± 1.60^Bb^	55.30 ± 0.57^ABbc^	6.55 ± 0.20^ABb^	0.20 ± 0.01^Aab^	0.74 ± 0.01^Bbc^
	TTCG (135/1035)	1766.67 ± 19.95^Ccd^	16.22 ± 0.09^CDc^	97.64 ± 1.63^Bb^	51.02 ± 0.67^Bc^	8.58 ± 0.16^Aa^	0.04 ± 0.01^Bc^	0.78 ± 0.01^Bbc^
	TTGG (45/1035)	1550.00 ± 30.77^De^	15.53 ± 0.16^Ed^	87.00 ± 3.17^Bb^	61.24 ± 1.27^Aa^	8.30 ± 0.16^Aa^	0.20 ± 0.01^Aab^	0.88 ± 0.01^Ca^

Different lowercase letters indicate significant differences (p < 0.05); different uppercase letters indicate highly significant differences (p < 0.01). Sample sizes were limited for several haplotypes (n < 20).

### Haplotype effects on slaughter traits

Haplotype means ± standard error for slaughter performance traits are presented in Table 18. In bucks, TTCC was the superior haplotype for live weight before slaughter, carcass weight, and back fat thickness. In does, CTCC demonstrated consistent superiority across multiple slaughter traits.

**Table 18:** Effects of haplotype on slaughter traits in Liaoning cashmere goat (mean ± standard error).

Name	Haplotype	Live weight before slaughter (kg)	Carcass weight (kg)	Net meat weight (kg)	Slaughter rate (%)	Net meat consumption rate (%)	Net carcass meat percentage (%)	Eye muscle area (cm²)	GR value (mm)	Back fat thickness (mm)
Buck	CCCC (4/32)	46.80 ± 0.30^ABab^	22.00 ± 0.25^b^	17.40 ± 0.06^ab^	47.01 ± 0.11^Bb^	37.18 ± 0.20	79.09 ± 0.25^ABa^	19.65 ± 0.70^b^	4.28 ± 0.62^Bb^	1.06 ± 0.12^Bb^
	CTCC (8/32)	41.55 ± 0.21^Bb^	21.75 ± 0.13^b^	16.25 ± 0.09^b^	52.34 ± 0.06^Aa^	39.11 ± 0.03	74.71 ± 0.02^Bb^	20.88 ± 0.94^ab^	7.94 ± 0.09^Aa^	1.63 ± 0.18^Bb^
	TTCC (12/32)	51.13 ± 2.07^Aa^	26.47 ± 1.61^a^	21.30 ± 1.53^a^	51.28 ± 1.36^ABa^	41.09 ± 1.72	79.76 ± 1.31^Aa^	19.57 ± 0.66^b^	5.80 ± 0.82^ABab^	3.18 ± 0.36^Aa^
	TTCG (8/32)	49.60 ± 1.29^Aa^	26.05 ± 0.36^a^	20.45 ± 0.36^a^	52.64 ± 0.64^Aa^	41.29 ± 0.35	78.47 ± 0.30^ABa^	24.02 ± 1.66^a^	7.16 ± 0.57^ABa^	1.79 ± 0.04^Bb^
Doe	CTCC (6/72)	57.50 ± 0.53^Aa^	26.20 ± 0.45^Aa^	21.20 ± 0.26^Aa^	45.57 ± 0.05^Bc^	36.87 ± 0.15^Bb^	80.92 ± 0.35^ABbc^	20.93 ± 0.46^Aab^	6.39 ± 0.09^Cc^	1.65 ± 0.18^Bc^
	CTCG (12/72)	47.45 ± 0.59^Bb^	23.50 ± 0.27^ABb^	19.50 ± 0.33^ABa^	49.53 ± 0.04^Ab^	41.07 ± 0.19^Aa^	82.92 ± 0.45^Aa^	20.45 ± 0.35^Aab^	7.84 ± 0.07^Bb^	2.85 ± 0.12^Aab^
	CTGG (12/72)	40.75 ± 0.80^Cc^	20.35 ± 0.35^Cc^	16.85 ± 0.38^Bb^	49.97 ± 0.13^Ab^	41.32 ± 0.11^Aa^	82.71 ± 0.44^Aa^	16.43 ± 0.56^Bc^	6.43 ± 0.16^Cc^	2.50 ± 0.06^ABb^
	TTCC (30/72)	47.72 ± 1.03^Bb^	24.38 ± 0.71^Aba^	20.16 ± 0.67^Aa^	50.89 ± 0.40^Aab^	41.98 ± 0.49^Aa^	82.45 ± 0.31^Aab^	19.33 ± 0.24^Ab^	9.72 ± 0.17^Aa^	3.32 ± 0.21^Aa^
	TTCG (12/72)	40.55 ± 0.80^Cc^	20.90 ± 0.60^BCc^	16.70 ± 0.12^Bb^	51.73 ± 0.87^Aa^	41.43 ± 1.11^Aa^	79.93 ± 0.81^Bc^	21.25 ± 1.18^Aa^	6.43 ± 0.53^Cc^	2.58 ± 0.62^Ab^

Different lowercase letters indicate significant differences (p < 0.05); different uppercase letters indicate highly significant differences (p < 0.01). Sample sizes were limited for several haplotypes (n < 20).

## DISCUSSION

### Population genetic characteristics of the C31799T and C34197G loci

This deviation is likely attributable to multiple factors. Primarily, the long-term, intensive artificial selection practiced to improve CF and growth performance has likely led to directed changes in allele frequencies and the evolution of the population’s genetic structure [17]. Additionally, factors such as the limited size of the breeding population, skewed sex ratios, or a certain degree of inbreeding may have exacerbated this deviation [18]. In our statistical analyses, we accounted for these potential confounding factors by incorporating pedigree information (to control for kinship), and by including sex and age as fixed effects in a general linear model. This approach was implemented to mitigate the interference of these factors on the detected association signals.

### Potential biological roles of *GMPS* in CF

*GMPS* and *SRC* are evolutionarily conserved genes with pivotal biological functions across species. *GMPS* functions as a key rate-limiting enzyme in the purine synthesis pathway, responsible for catalyzing the production of guanosine monophosphate (GMP), thereby supplying essential substrates for cellular proliferation. Given the high metabolic demand during hair follicle growth and cyclic regeneration, we speculate that *GMPS* may influence CF by modulating the proliferative activity of follicular keratinocytes. Although initial functional characterization was primarily conducted in model organisms such as bacteria, yeast, and insects [19–21], emerging evidence reveals complex regulatory capacities beyond canonical enzymatic activity, and subsequent studies have substantiated its significant role in tumor biology. Notably, *GMPS* has been identified as a critical molecular hub that integrates intracellular mechanisms with extracellular microenvironmental signals across various cancers. Building upon these findings, we propose that *GMPS* may regulate CF in LCGs through a dual mechanism: (1) as a metabolic enzyme providing purine nucleotides for rapidly dividing follicle cells during fiber synthesis, and (2) as a signaling modulator potentially involved in follicular microenvironment communication, analogous to its role in tumor-stroma interactions.

### Potential biological roles of *SRC* in CF and slaughter performance

On the other hand, the *SRC* family exerts central regulatory functions in core physiological processes, including reproduction, metabolism, and the stress response, by significantly enhancing the transcriptional activity of various steroid hormone receptors [22]. It is crucial to distinguish this *SRC* coactivator from the *S*FKs, which are non-receptor tyrosine kinases involved in intracellular signal transduction [4]; this study focuses on the former. Steroid hormones (e.g., estrogen, progesterone) are key regulators of hair follicle development and cycle. We hypothesize that *SRC*, as a universal transcriptional coactivator, may amplify localized hormonal signaling within skin follicles, thereby modulating the proliferation and differentiation of follicular stem cells, ultimately influencing secondary follicle density and CF. Substantial evidence supports its vital role in development; for instance, *SRC1* and *SRC2* act synergistically to regulate extracellular matrix remodeling and multi-organ development, which is essential for ensuring normal fetal lung maturation, a critical signal event for initiating parturition. Furthermore, *SRC* is highly likely to mediate progesterone-dependent transcription of genes like *Crispld2* during lung development [23]. By analogy, *SRC* may orchestrate gene expression related to cashmere follicle growth and fiber synthesis in a similar manner. Consequently, polymorphisms in *SRC* might concurrently affect cashmere traits and slaughter performance by modulating hormone responsiveness and associated metabolic pathways. This viewpoint finds indirect support in ovine studies confirming that *SRC* is an essential molecular switch that enables glucocorticoids to exert their organ-protective effects, thereby precisely coordinating hormonal signaling and gene expression [24, 25].

### Pleiotropic effects and sex-specific genetic regulation of production traits

*GMPS* and *SRC* are hub genes regulating critical life processes, with well-defined functions in human medicine and fundamental physiology. This study focused on their roles in LCGs. Through SNP genotyping and association analysis, we found that polymorphisms at the *GMPS* C31799T and *SRC* C34197G loci not only significantly influenced CF but also exhibited pleiotropic effects on other production traits. The superior genotypes displayed distinct and sex-dependent patterns that may reflect a resource-allocation trade-off between fiber synthesis and muscle growth, coordinated by pleiotropic genes. For instance, the T allele of the *GMPS* gene might preferentially direct metabolic resources toward cashmere production, resulting in finer fibers, whereas the C allele appears more beneficial for body growth and muscle deposition. Similarly, the C allele of the *SRC* gene seems to contribute positively to both CF and meat quality. Path analysis further elucidated these complex relationships. In bucks, net meat weight exhibited a significant positive direct effect on fiber diameter (direct path coefficient: 31.82), while net meat rate showed the strongest negative direct effect (–29.92), revealing a genetic antagonism between meat and cashmere traits. In does, both slaughter rate and carcass net meat percentage demonstrated consistent negative direct effects (–1.05 and –0.52, respectively), and their indirect effect pathways were entirely different from those in bucks. This supports the argument for sex-specific genetic regulation underlying the correlation of these traits. In summary, these results suggest that *GMPS* and *SRC* may co-regulate key economic traits such as CF, body size, and slaughter performance by coordinating resource allocation between follicular development and muscle metabolism, within a sex-specific genetic regulatory architecture.

### Comparison with genetic studies in other cashmere goat breeds

The effects of *GMPS* and *SRC* genes identified in the LCG in this study show both commonalities and distinctions compared to findings in other cashmere goat breeds. Population genetics studies indicate genetic differentiation between LCG and some other local Chinese breeds [26], suggesting that the genetic architecture underlying CF may exhibit breed specificity. The key candidate genes influencing fineness appear to differ across breeds. For instance, in Shaanbei White Cashmere goats, polymorphisms in genes such as *Notch2* have been significantly associated with cashmere growth and body size traits [27]. Conversely, in Inner Mongolia Cashmere goats, the primary genetic determinants of fineness may involve different loci. Polymorphisms in the prolactin receptor (*PRLR*) gene have been linked to CF [28], while variations in the keratin-associated protein *KAP8.1* gene predominantly affect cashmere yield without a significant impact on fineness [29]. These findings collectively suggest that the key genes regulating CF in Inner Mongolia Cashmere goats are likely distinct from the *GMPS* and *SRC* genes identified in our study. Furthermore, recent genomic analyses of Tibetan Northwest White Cashmere goats reveal that their finer cashmere fibers are associated with specific genomic regions under strong selection, which differ significantly from those in plain-breed cashmere goats [30]. These comparisons underscore the diverse genetic foundations of key economic traits in cashmere goats. Our study is the first to report significant associations between *GMPS*/*SRC* polymorphisms and CF in LCG, thereby contributing novel molecular markers to the expanding toolkit for molecular breeding in cashmere goats.

### Correlations among cashmere production and slaughter traits

The present study demonstrates a significant correlation between cashmere yield and cashmere length, with the greatest indirect effect coefficient being 0.318. Through research conducted by Feng *et al*. [31] on Shaanbei White Cashmere goats, it was found that the correlation coefficient between cashmere yield and cashmere length was the highest, followed by chest circumference, body weight, and cannon circumference. Studies by He Xiaohong *et al*. [32] indicated that cashmere length had the greatest impact on cashmere yield, chest circumference had the strongest influence on body weight, while body slope length, cannon circumference, and staple length indirectly affected body weight through chest circumference. Research by Li Longping *et al*. [33] revealed extremely significant positive correlations among cashmere yield, cashmere length, CF, and body weight in Shaanbei White Cashmere goats, with interactions observed among these traits (cashmere length, fineness, body weight, etc.). Based on the aforementioned literature, we hypothesized whether traits correlated with CF exist within both cashmere production and slaughter performance. Our correlation analysis between CF and slaughter traits revealed significant correlations between CF and slaughter rate, net meat rate, net meat percentage of carcass, and eye muscle area. Furthermore, correlation analysis between CF and various traits showed significant correlations between CF and cashmere yield, cashmere length, coefficient of variation of length, number of crimps, short fiber content, and cashmere rate. These findings are consistent with reports by Shi Lei *et al*. [34], who found a highly significant positive correlation between CF and length, and Zhang Fuquan *et al*. [35], who reported a positive correlation between CF and yield. This suggests that the physiological processes that control fiber fineness may be genetically or metabolically coupled to muscle development, although the specific mechanisms of interaction require further investigation.

### Implications for molecular breeding and future research directions

This study reveals statistically significant associations rather than direct causal relationships. Nonetheless, the identified superior genotypes and haplotype combinations provide valuable pre-selection markers for molecular breeding in LCGs. The observed negative correlation between CF and slaughter traits may stem from pleiotropy, tight genetic linkage, or indirect effects of physiological resource allocation, constituting a key focus for subsequent functional studies to elucidate the specific molecular interactions. In practical breeding, where the core objectives are to consistently reduce fiber diameter, each micrometer reduction can significantly enhance market value, while maintaining high yield, we propose a MAS strategy involving: 1) the establishment of sex-specific selection protocols, and 2) the development of criteria for early genetic evaluation and retention of replacement breeding stock based on the significant effects of *GMPS* and *SRC*. Validation of these markers in independent populations is expected to improve selection accuracy for fineness, shorten the genetic improvement cycle, and thus enable a steady reduction in fiber diameter while preserving cashmere yield. Future work should prioritize validation in larger populations to confirm stability, followed by research to identify key causal mutations and clarify the expression mechanisms of these genes within hair follicles, thereby strengthening the theoretical foundation and technical support for future breeding programs.

## CONCLUSION

This study is the first to establish significant associations between polymorphisms in *GMPS* and *SRC* and CF as well as multiple production traits in LCG. The TT genotype at the *GMPS* C31799T locus and the CG genotype at the *SRC* C34197G locus were significantly linked to finer cashmere fibers (p < 0.05). Sex-stratified analyses revealed clear sexual dimorphism: the *GMPS* TT genotype conferred superior CF in does (p < 0.01) and longer staple length in both sexes (p < 0.01), whereas the *SRC* CG genotype improved fineness specifically in does (p < 0.01). Haplotype analysis identified CCCC as the optimal combination for finer cashmere in bucks and TTGG in does. Pleiotropic effects were evident across traits: the *GMPS* CC genotype favored larger body dimensions, the *GMPS* TT genotype enhanced carcass traits in bucks, and the *SRC* CG genotype improved lactation performance in does. Path and stepwise regression analyses confirmed sex-dependent genetic trade-offs, with CF showing positive correlations with cashmere yield (bucks: r = 0.412; does: r = 0.384; p < 0.01) and negative associations with several slaughter traits.

The superior genotypes (*GMPS* TT and *SRC* CG) and haplotypes (CCCC in bucks, TTGG in does) identified in this study represent immediately deployable, sex-specific molecular markers for MAS in LCG breeding programs. Their routine implementation will accelerate genetic progress toward premium fiber diameters (<16 μm) while maintaining meat yield and quality through balanced multi-trait selection indices. Early genotyping of replacement breeding stock will reduce the generation interval, increase selection accuracy, and deliver substantial economic benefits to cashmere goat producers in Liaoning Province and comparable production systems.

The investigation was performed on a large, well-managed nucleus herd (n = 1 160) with comprehensive phenotyping of cashmere production traits, body size measurements, slaughter performance, meat quality attributes, milk composition, and lambing rate. Rigorous statistical approaches, including general linear mixed models with pedigree-based relatedness control, sex-stratified haplotype inference, path analysis, and stepwise regression, provided robust adjustment for kinship, age, and sex effects. The combination of functional biological knowledge of *GMPS* and *SRC* with population-level association data strengthens the interpretability and biological plausibility of the results.

The associations were detected within a single nucleus population and therefore require independent validation in additional LCG herds and, ideally, in other cashmere goat breeds to confirm wider applicability. The identified SNPs are intronic; their exact functional consequences (e.g., on splicing efficiency, regulatory element activity, or gene expression) remain to be elucidated. Although major environmental and management factors were standardized and controlled, residual population stratification or unmeasured gene–environment interactions cannot be completely ruled out.

Priority should be assigned to (1) functional validation of the candidate SNPs through *in vitro* reporter assays, CRISPR-based editing, and follicle-specific expression profiling; (2) fine-mapping and causal variant identification using high-density SNP arrays or whole-genome resequencing; (3) multi-herd and multi-breed validation studies to evaluate marker stability and transferability; (4) integration of the *GMPS* and *SRC* markers into genomic prediction models for simultaneous improvement of fiber quality and meat traits; and (5) examination of gene–nutrition and gene–management interactions to refine precision breeding recommendations for LCG.

By identifying *GMPS* and *SRC* as novel candidate genes regulating CF in LCG, this research provides a robust molecular basis for sex-specific MAS. The superior genotypes and haplotypes reported here constitute powerful, immediately usable tools for accelerating genetic improvement in fiber quality while preserving the breed’s dual-purpose productivity. Widespread adoption of these markers, combined with continued functional genomics and validation studies, will support sustainable enhancement of cashmere value and strengthen the long-term economic competitiveness of cashmere goat production in China and beyond.

## DATA AVAILABILITY

All the generated data are included in the manuscript.

## AUTHORS’ CONTRIBUTIONS

QY: Formal data analysis, interpretation and drafted and revised the manuscript. RD and QZ: Study design and experimental implementation, data collection, and final manuscript revision. SL, LK, YZ, HL, HM, WH, WL, HW, XK, DL, YG, and JL: Collected and pretreated experimental samples (core step of research data collection), verified experimental data, and final manuscript review. ZW: Conceived and designed the study, developed core research methodologies, supervised the entire process of experiments and data analysis, and revised and finalized the manuscript. All authors have read and approved the final version of the manuscript.
